# Epigenetic reprogramming of breast cancer cells with oocyte extracts

**DOI:** 10.1186/1476-4598-10-7

**Published:** 2011-01-13

**Authors:** Cinzia Allegrucci, Michael D Rushton, James E Dixon, Virginie Sottile, Mansi Shah, Rajendra Kumari, Sue Watson, Ramiro Alberio, Andrew D Johnson

**Affiliations:** 1 Centre for Genetics and Genomics, School of Biology, University of Nottingham, Queens Medical Centre, Nottingham, NG2 2UH, UK; 2 School of Veterinary Medicine and Science, University of Nottingham, Sutton Bonington Campus, Loughborough, LE12 5RD, UK; 3 School of Clinical Sciences, Wolfson Centre for Stem Cells, Tissue Engineering and Modelling, University of Nottingham, University Park, NG7 2RD, UK; 4 School of Clinical Sciences, Division of Preclinical Oncology, University of Nottingham, Queens Medical Centre, Nottingham, NG2 2UH, UK; 5 School of Biosciences, University of Nottingham, Sutton Bonington Campus, Loughborough, LE12 5RD, UK

## Abstract

**Background:**

Breast cancer is a disease characterised by both genetic and epigenetic alterations. Epigenetic silencing of tumour suppressor genes is an early event in breast carcinogenesis and reversion of gene silencing by epigenetic reprogramming can provide clues to the mechanisms responsible for tumour initiation and progression. In this study we apply the reprogramming capacity of oocytes to cancer cells in order to study breast oncogenesis.

**Results:**

We show that breast cancer cells can be directly reprogrammed by amphibian oocyte extracts. The reprogramming effect, after six hours of treatment, in the absence of DNA replication, includes DNA demethylation and removal of repressive histone marks at the promoters of tumour suppressor genes; also, expression of the silenced genes is re-activated in response to treatment. This activity is specific to oocytes as it is not elicited by extracts from ovulated eggs, and is present at very limited levels in extracts from mouse embryonic stem cells. Epigenetic reprogramming in oocyte extracts results in reduction of cancer cell growth under anchorage independent conditions and a reduction in tumour growth in mouse xenografts.

**Conclusions:**

This study presents a new method to investigate tumour reversion by epigenetic reprogramming. After testing extracts from different sources, we found that axolotl oocyte extracts possess superior reprogramming ability, which reverses epigenetic silencing of tumour suppressor genes and tumorigenicity of breast cancer cells in a mouse xenograft model. Therefore this system can be extremely valuable for dissecting the mechanisms involved in tumour suppressor gene silencing and identifying molecular activities capable of arresting tumour growth. These applications can ultimately shed light on the contribution of epigenetic alterations in breast cancer and advance the development of epigenetic therapies.

## Background

Tissue homeostasis depends on tightly regulated mechanisms controlling cell proliferation and differentiation. Expression of proto-oncogenes and tumour suppressor genes controls normal cell function, and misregulation of these genes by both genetic and epigenetic alterations is at the origin of cancer [1,2]. Genetic changes include deletion, mutation and amplification of genes, whereas epigenetic alterations occur without change in DNA sequence via modification of chromatin organisation, including DNA methylation, histone modifications and expression of non-coding RNAs. The role of epigenetic alterations in tumourigenesis has been recognised in different types of malignancies, including breast cancer [1].

In the breast, abnormal epigenetic regulation of genes regulating the cell cycle, apoptosis, DNA repair, cell adhesion and signalling leads to tumour formation, its progression, and drug resistance [3]. Epigenetic alterations prevail over genetic abnormalities in initial stages of breast tumour development. For instance, silencing of *CDKN2A *(*p16INK4A*), *HOXA *and *PCDH *gene clusters by DNA methylation together with over-expression of Polycomb proteins BMI-1, EZH2 and SUZ12 occurs during spontaneous or induced transformation of human mammary epithelial cells [4,5]. Methylation of several homeobox genes is also observed in ductal carcinoma *in situ *and stage I breast tumours [6].

Unlike genetic alterations, epigenetic modifications of the chromatin are reversible and therefore are suitable targets for reversal or attenuation of malignancy. The question of how tumours can be reprogrammed is intriguing, and determining how a cancer cell can be reprogrammed back to a normal cell phenotype is important not only for understanding the molecular pathways of the disease but also for diagnostic and therapeutic intervention [7].

Embryonic environments that program cell fate during development are able to reverse tumorigenicity [8]. Landmark experiments have shown that teratocarcinoma cells are reprogrammed when injected into a mouse blastocyst resulting in normal tissue derived from tumour cells in chimeric mice [9]. Tumorigenicity of metastatic melanoma cells is also reduced when cells are injected into zebrafish [10], chicken [11] and mouse embryos [12] or when they are cultured on 3D-matrices conditioned with human embryonic stem cells [13].

Nuclear transfer (NT) experiments have demonstrated that oocytes can fully reset the epigenotype of somatic cells [14] and this ability has been exploited to re-establish developmental potential in teratocarcinoma, medulloblastoma and melanoma cells to extents that depend on the degree of non-reprogrammable karyotypic abnormalities of the donor tumour cell nucleus [15-17]. Because NT experiments depend on the ability of reprogrammed cells to support embryonic development, with either formation of viable offspring or blastocyst-derived embryonic stem cells as potential outcomes, they are not easily amenable to dissecting the molecular mechanisms involved in tumour reversion. Understandably, NT experiments also do not allow the study of human tumour cells.

An alternative method to reprogram cells is using oocyte extracts as an *ex-ovo *system [18]. Extracts made from amphibian oocytes are of particular interest, since they are available in large quantities and they possess reprogramming abilities similar to those of mammalian oocytes [19-22]. We have previously shown that amphibian oocyte extracts possess activities able to modify DNA methylation and histone marks, together contributing to the remodelling of somatic cell chromatin [21,23]. In addition, we have introduced oocytes from axolotls, a urodele (salamander) amphibian, as a source of reprogramming extract based on our previous demonstrations that urodeles are genetically more similar to mammals and the molecular mechanisms governing the early development of urodeles and mammals are conserved [24-28]. In this study we analyse the relative efficiencies of extracts from oocytes of axolotl and *Xenopus *for their ability to reverse epigenetic alterations within breast cancer cell chromatin. Our results show that axolotl oocyte extracts reprogram cancer cell chromatin with high efficiency, reversing epigenetic silencing and activating expression from tumour suppressor genes whose repression is involved in breast tumorigenesis. In addition, we show long term suppression of tumour growth *in vivo *by reprogramming with oocyte molecules.

## Results and Discussion

### Oocyte extracts induce expression of silenced tumour suppressor genes

Understanding the molecular mechanisms involved in loss of tumour suppressor gene function represents a major hurdle for cancer therapies, towards which reversion of cancer cell tumorigenicity can provide important clues. In this study we asked whether epigenetically silenced tumour suppressor genes could be reprogrammed to a transcriptionally active state by the chromatin remodelling activities present in oocyte extracts. A panel of genes known to be silenced in breast cancers were selected to address this question. These genes were either not expressed or were expressed at very low levels in MCF-7 and HCC1954 cell lines, representing luminal and basal breast cancer phenotypes, respectively (Table 1).

**Table 1:** Expression of tumour suppressor genes in reprogrammed breast cancer cells

Gene	MCF-7	MCF-7 in AOE	HCC1954	HCC1954 in AOE	HMEC
*RARB*	-	**+**	-	**+**	**++**

*CST6*	-	**+**	-	**+**	**+**

*CCND2*	-	**+**	-	**+**	**+++**

*GAS2*	-	**+**	-	**+**	**+++**

*ST18*	-	-	-	-	**+**

*SRBC*	-	-	-	-	**+**

*SCGB3A1*	-	-	-	-	**+**

*RASSF1A*	-	-	-	-	**+**

*GSTP1*	-	-	-	-	**+**

*CDKN2A*	-*	n/a	-	**+**	**+**

* = deleted in MCF-7; n/a = not applicable

**Figure 1:**
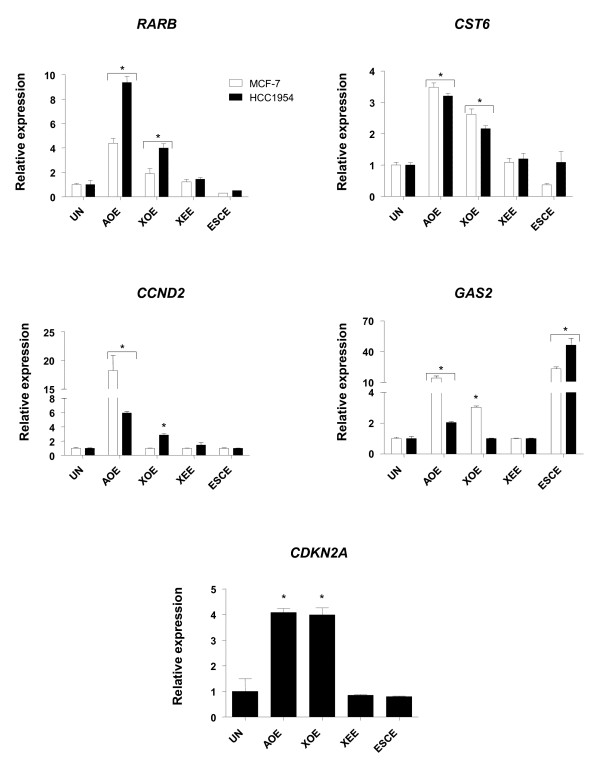
**Expression of tumour suppressor genes after reprogramming in oocyte, egg and embryonic stem cell extracts **Expression of *RARB*, *CST6*, *CCND2*, *GAS2 *and *CDKN2A *after 6 hours reprogramming analysed by Q-PCR. Data are shown as fold increase compared to the calibrator sample (UN: untreated cells). Relative quantification to the expression of *ACTIN *(*ACTB*) was performed for each gene. Study of *CDKN2A *expression of was only performed in HCC1954 since this gene is deleted in MCF-7 cells.* indicates P < 0.05 for treated groups different from UN.

We have previously shown that oocytes of two amphibian species (*Xenopus laevis *and *Ambystoma mexicanum *or axolotl) are able to induce chromatin remodelling in somatic cells [21,23], and therefore we tested whether extracts made from prophase oocytes could alter epigenetic marks of cancer cells. Digitonin treatment of breast cancer cells gently permeabilises cellular and nuclear membranes, as demonstrated by their cytosolic permeability to 70KDa FITC-dextran and their viability after extract treatment (Additional file 1: Figure S1). Our previous work shows that chromatin remodelling occurs within 3 to 6 hours when fibroblasts are incubated in oocyte extracts [21,23], so we chose the longest time point to assess reprogramming of tumour suppressor genes. Both axolotl and *Xenopus *oocyte extracts (AOE and XOE, respectively) were able to induce re-expression of *RARB*, *CST6*, *CCND2*, *GAS2 *and *CDKN2A *genes. Importantly, re-expression of *RARB*, *CST6*, *CCND2 *was observed in both breast cancer cell lines (*CDKN2A *expression was only investigated in HCC1954 cells since this gene is deleted in MCF-7) (Figure 1). The reprogramming activity of AOE was greater than XOE for all genes with the exception of *CDKN2A*, which is induced to the same extent by either extract. The relative level of induced gene expression varied between the two cell lines; however, not all the reprogrammed tumour suppressor genes were re-expressed at levels similar to those of normal human mammary epithelial cells (HMEC). Also, some of the genes analysed (*ST18*, *SRBC*, *SCGB3A1*, *RASSF1A*, *GSTP1*) did not alter their expression in response to extract treatment (Table 1). The fact that only a sub-set of the tested tumour suppressor genes were re-activated after treatment suggests that some genes may be more susceptible to reprogramming than others. Indeed, silencing of cancer-related genes is mediated by epigenetic modifications encompassing wide genomic regions [29], so it is possible that reprogramming of some tumour suppressor genes may depend on their genomic context. Alternatively, factors required for transcriptional activation might not be present, and/or the time of incubation may not have been sufficient to induce re-expression of all silenced domains. Nevertheless, the data clearly demonstrate that re-activation of tumour suppressor genes is highly specific and restricted to activities contained in prophase oocytes, as no effect was observed when cells were treated with matured (Metaphase II) *Xenopus *egg extracts (XEE) (Figure 1). Because eggs are transcriptionally inert, these results suggest that transcriptional activation is induced by factors present in oocytes [21]. Further, oocyte extracts sustain binding of TATA-binding protein to transcriptionally active promoters [20], and we have previously demonstrated that amphibian extracts restore Polymerase II transcription in mammalian cells incubated at amphibian compatible temperatures (17°C). These results suggest that during the incubation period transcription is directed by amphibian molecules [21].

We next compared the reprogramming capacity of these extracts with extracts prepared from embryonic stem cells (ESC), as it has been reported that ESC extracts (ESCE) can also reprogram transcription of somatic cells to pluripotency [20,23,30]. Mouse ESCE were used so that we could control for activation of human genes in this mammalian heterologous system; reprogramming was performed at mammalian physiological temperature (37°C). Surprisingly, ESCE only induced expression of *GAS2 *(Figure 1). Epigenetic silencing of tumour suppressor genes involves diverse mechanisms, including DNA methylation, histone modifications and expression of non coding RNAs [2]. It is therefore possible that activities contained in oocyte and ESC extracts can differentially reprogram these epigenetic marks. The limited reprogramming capacity of ESCE that we demonstrate here is in agreement with a previous report in which changes in tumour suppressor gene expression were not observed when teratocarcinoma cells were used to reprogram somatic cells to pluripotency [31]. Taken together, our results highlight intrinsic differences in the reprogramming ability of oocytes and ESC for tumour suppressor genes, which we believe extend from the natural chromatin remodelling activities found in oocytes [18].

Intriguingly, AOE was consistently more efficient in re-activating the majority of silenced genes compared with the activities in XOE. It is important in this regard to note that axolotl oocytes were chosen for this study for the specific reason that urodele amphibians reflect the ancestral amphibian state from which mammals evolved, and they are therefore more genetically similar to mammals than are frogs [25-28]. As a consequence, transcription factor compatibility with mammalian target sequences would be expected to be greater, and axolotl oocytes would more closely reflect the epigenetic remodelling activity of mammalian oocytes [25-28], (ADJ, unpublished). Hence, AOE provides a powerful tool to identify mechanisms that mediate the reversal of epigenetic silencing of tumour suppressor genes involved in human cancers.

### Demethylation of tumour suppressor gene promoters by oocyte extracts

**Figure 2:**
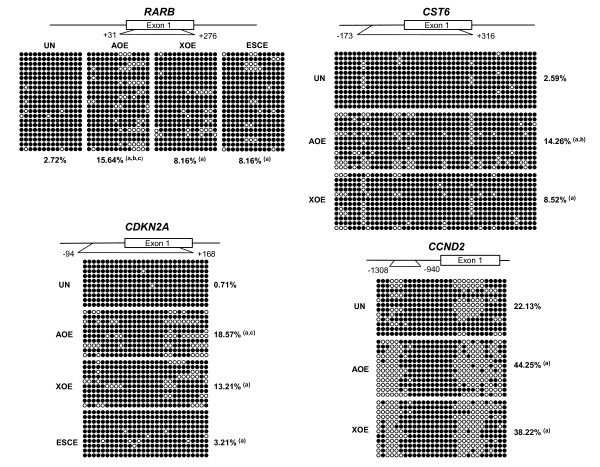
**DNA methylation analysis of tumour suppressor genes by bisulfite sequencing**. Bisulfite sequencing of *RARB*, *CST6*, *CCND2 *(MCF-7 cells), and *CDKN2A *(HCC1954 cells) gene promoters after 6 hours reprogramming. Schematics indicate the position of analysed CpG islands in promoter regions. A minimum of 10 clones were analysed for each gene and average loss of methylation was calculated for each reprogramming treatment. Black circles indicate metylated CGs, white circles indicate unmethylated CGs. Reprogramming in AOE produced the highest levels of demethylation (P < 0.05; a = AOE, XOE, ESCE vs UN; b = AOE vs XOE; c = AOE vs ESCE).

Amphibian oocytes possess replication independent DNA demethylating activity and they can reduce DNA methylation at a genome-wide level as well as at pluripotency gene promoters in mammalian cells [22,23]. Because epigenetically silenced tumour suppressor genes are generally hypermethylated in breast cancer, we next sought to determine whether the re-activation of silenced genes was due to a reduction of DNA methylation at promoter regions. Bisulphite sequencing shows demethylation of tumour suppressor gene promoters after exposure to AOE and XOE, when compared to untreated control cells (P < 0.05, Figure 2). AOE induced higher levels of demethylation for *RARB *and *CST6 *promoters compared to XOE (P < 0.05), but *CDKN2A *and *CCND2 *showed similar levels of demethylation by either extract. This result correlates with the greater efficiency of AOE in inducing gene expression of *RARB *and *CST6 *compared to XOE, whereas *CDKN2A *expression is induced to similar levels by either extract. However, this does not explain the greater induction of *CCDN2 *expression by AOE. Interestingly, ESCE induced limited demethylation of *RARB *and *CDKN2A *genes, which is consistent with the inability of this extract to re-activate their expression. Recent work indicates that active DNA demethylation in oocytes is controlled by base excision repair [32], and these mechanisms might be less active in ESC. Consistent with gene expression results, we did not observe DNA demethylation in the promoters for two of the tumour suppressor genes that were not reprogrammed by oocytes extracts, *RASSF1A *and *ST18 *(data not shown). We do not interpret this result as a limited activity of the oocyte extracts, but rather as a reflection of the refractory nature of some silenced promoters to demethylation under these experimental conditions.

Importantly, extract-induced demethylation at promoter regions was not randomly distributed among CpG dinucleotides. Demethylation of CGs number 8-13 was found for *RARB*, CGs 1-8, 14-18, 25 and 31 for *CST6*, CGs 1-7 and 21-28 for *CCND2*, and CGs 1-9 and 19-28 for *CDKN2A*. Interestingly, the majority of these demethylated CpG residues contain putative Sp1 sites (Table 2), suggesting that DNA methylation mediated by oocyte and ESC extracts is driven specifically at these genomic regions to reactivate transcription. Reprogramming experiments with somatic cells in *Xenopus *oocytes support these conclusions. For example, re-activation and demethylation of the *Oct-4 *gene promoter, after injection of thymocytes into *Xenopus *oocytes, is strictly dependent on the presence of a Sp1 site in its promoter [22]. Although it is well established that the transcription factor Sp1 can provide protection of housekeeping genes from CpG island methylation [33,34], it is remarkable that this site can be targeted specifically for demethylation in the promoters of tumour suppressor genes, with high efficiency, by DNA demethylating complexes present in the extracts. It will now be interesting to identify how targeted demethylation is regulated.

**Table 2:** Sp1 sites in demethylated tumour suppressor gene promoters

	*RARB*	*CST6*	*CCND2*	*CDKN2A*
Number of demethylated CGs	8-13	1-8, 14-18, 25, 31	1-7, 21-28	1-9, 19-28

Number of demethylated CGs containing Sp1 sites	12	5, 12, 13	1, 2	9, 10, 22

Putative transcription factors binding sites contained in demethylated CGs	NF-kappaB, c-Re1	GATA-1, Ahr/Arnt	CREB, MYC, USF1, MAX, SRY, MZF-1	USF1, MZF-1, CP2

Putative transcription factor binding sites as determined by TFSEARCH http://www.cbrc.jp/research/db/TFSEARCH.html.

Previous work demonstrates that amphibian oocytes induce expression of pluripotency genes in somatic cells [22,23]. As re-expression of these genes in cancer cells may induce an adverse cancer stem cell phenotype, we investigated whether AOE can induce demethylation of *OCT-4 *and *NANOG *gene promoters, as well as expression of the respective proteins. After 6 hours of reprogramming and 6 days of culture, we observed no change in DNA methylation at pluripotency gene promoters (Additional file 2: Figure S2A). Consistently, OCT-4 was not expressed after 6 days; however we detected NANOG protein expression in untreated and treated cells, likely as result of cross-reactivity of the NANOG antibody with the protein encoded by the *NANOGP8 *pseudogene (Additional file 2: Figure S2B). Cancer cells predominantly express *NANOG *transcripts derived from the *NANOGP8 *retrogene and because the protein encoded by the pseudogene is almost identical to the native NANOG protein, it can be easily recognised by anti-NANOG antibodies [35].

### Remodelling of histone marks by oocyte extracts

**Figure 3:**
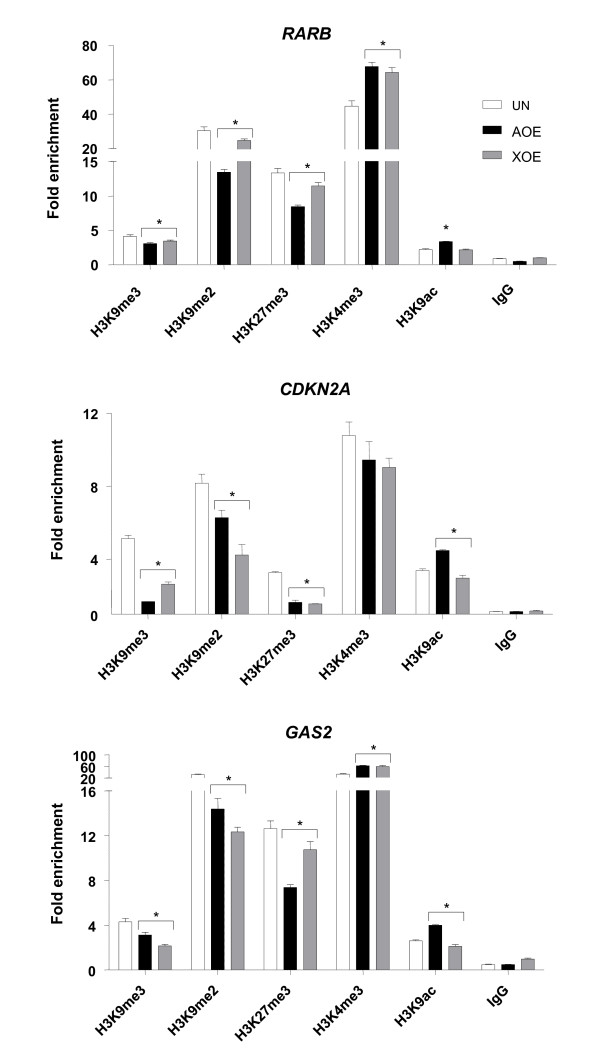
**Reprogramming of histone marks by oocyte extracts**. Analysis of *RARB*, *CDKN2A *and *GAS2 *gene promoters by ChIP. Data are presented as fold enrichment to input chromatin and indicate reprogramming of histone repressive (H3K9me3, H3K9me2, H3K27me3) and active (H3K4me3, H3K9Ac) marks by different extracts after 6 hours of treatment. * indicates P < 0.05 for treated groups different from UN.

We next investigated the remodelling of histone marks at re-activated gene promoters, in order to establish the relationship between this epigenetic modification and DNA demethylation in breast cancer. We focussed our attention on the *RARB*, *CDKN2A *and *GAS2 *promoters (Figure 3). Chromatin immunoprecipitation (ChIP) shows that AOE and XOE reduce transcription-repressive histone marks, such as trimethylation of histone H3 at lysine 27 (H3K27me3), trimethylation of histone H3 at lysine 9 (H3K9me3), and dimethylation of histone H3 at lysine 9 (H3K9me2). Trimethylated lysine 4 at histone H3 (H3K4me3), representing a transcription-active mark, was modified at *RARB *and *GAS2 *promoters, but not at the *CDKN2A *promoter. H3K4me3 was found in each of the promoters we analysed, suggesting that silencing results from the addition of repressive epigenetic marks. Interestingly, we observed a modest increase in acetylation of Histone H3 lysine 9 (H3K9ac), which may relate with the low levels of transcriptional activation obtained for some genes. Of all genes analysed, *GAS2 *was the only one to be re-activated by ESCE. Because *GAS2 *expression is not regulated by DNA methylation [36], we analysed the effect of ESCE on remodelling of histone marks in its promoter region. ESCE effectively reduced H3K9me3, H3K9me2 and H3K27me3 marks, supporting the observed gene activation (Additional file 3: Figure S3).

Taken together, our results show very effective epigenetic reprogramming of tumour suppressor gene promoters by oocyte extracts, encompassing DNA demethylation and reversion of histone marks to a more euchromatic state. By comparison, treatment with 5-aza-2'-deoxycytidine can demethylate DNA in the promoters of tumour suppressor genes, but H3K9me3 and H3K27me3, which characteristically mark heterochromatin, are often retained [37]. Most importantly, oocyte extracts induce expression from repressed tumour suppressor genes, and the higher level of expression induced by AOE than XOE correlates with a more robust targeting of demethylating activity in these extracts. Clearly, identifying the molecules that participate in the reprogramming of tumour suppressor gene expression could provide a route to the development of therapeutic strategies for the treatment of breast cancer.

### Stability of tumour suppressor gene reprogramming

**Figure 4:**
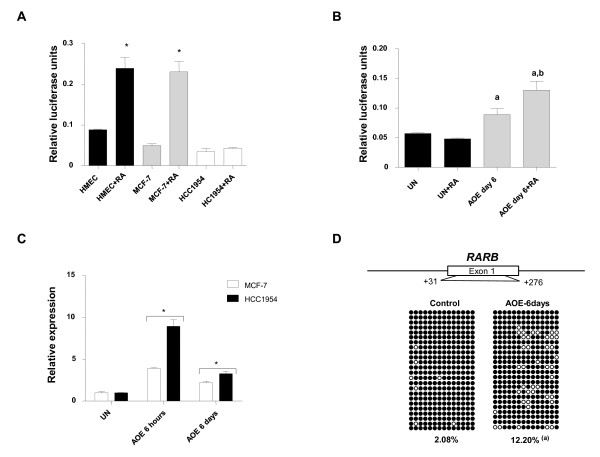
**Epigenetic reprogramming stability of tumour suppressor genes in AOE**. Reprogramming of the tumour suppressor gene *RARB *persists 6 days after treatment of MCF-7 and HC1954 cells with AOE. **(A) **Promoter activity in HMEC, MCF-7 and HCC1954 cells with or without RA treatment measured by luciferase assay (* indicates P < 0.05 for RA treated cells different from the untreated group). **(B) ***RARB *promoter in retinoic acid resistant HCC1954 cells can respond to RA after reprogramming (P < 0.05; a = AOE and AOE+RA vs UN; b = AOE + RA vs AOE). **(C) ***RARB *expression by Q-PCR. * indicates P < 0.05 of treated cells compared to UN. **(D) **DNA demethylation is maintained in HCC1954 cells after 6 days of treatment as shown by bisulfite sequencing (similar results were obtained with MCF-7 cells).

Because epigenetic reprogramming occurs within 6 hours of incubation in AOE, and in the absence of DNA replication, we sought to determine whether the epigenetic remodelling was stable. We specifically asked if the *RARB *gene remained active and responsive to retinoic acid (RA) treatment. We created transgenic cell lines carrying a *RARB *promoter fused to Firefly luciferase (*RARB-Lux*) to follow reprogramming over time. Silencing of the exogenous *RARB *promoter in MCF-7 and HCC1954 cell lines occurred rapidly, similar to previous studies [36]; and treatment with RA activated the reporter in responsive MCF-7 cells, but not HCC1954 cells (Figure 4A). In response to treatment with AOE, activity from the transfected *RARB *promoter was re-established in HCC1954 cells, and was maintained for at least 6 days (~5 population doublings) in culture (Figure 4B). In addition, expression from this promoter remained responsive to RA stimulation, indicating that stable reprogramming induced by AOE was maintained for the duration of the culture period. We confirmed this by showing that induced expression of the endogenous *RARB *gene was also maintained (Figure 4C), though the relative level of expression diminished over time. We then investigated the stability of demethylated CG residues by bisulphite sequencing. Consistent with the expression data, the results of these experiments show that the transcriptionally competent demethylated state of the *RARB *promoter was maintained, even though the cells had undergone several rounds of DNA replication (Figure 4D). Previous work indicates that the pluripotency gene *Oct-4 *undergoes gradual reprogramming after exposure to amphibian oocyte or egg molecules over 3-5 days, suggesting that replication-dependent reprogramming is necessary to reactivate some silenced genes [22,23,38]. However, this is not the case for *RARB; *furthermore those tumour suppressor genes that were not re-activated after 6 hours of AOE treatment, such as *RASSF1A *and *ST18*, remained silenced in long term culture (data not shown). Our results suggest that the maintenance of demethylated cytosine residues through the replication process is essential to long term reprogramming, which includes the responsiveness to inductive signals, such as RA. We view understanding how responsiveness to differentiation signals can be reprogrammed into the cancer cell genome as an important challenge with significant implications for therapeutic interventions.

### Reprogramming of tumour suppressor genes and reversal of malignant phenotype

**Figure 5:**
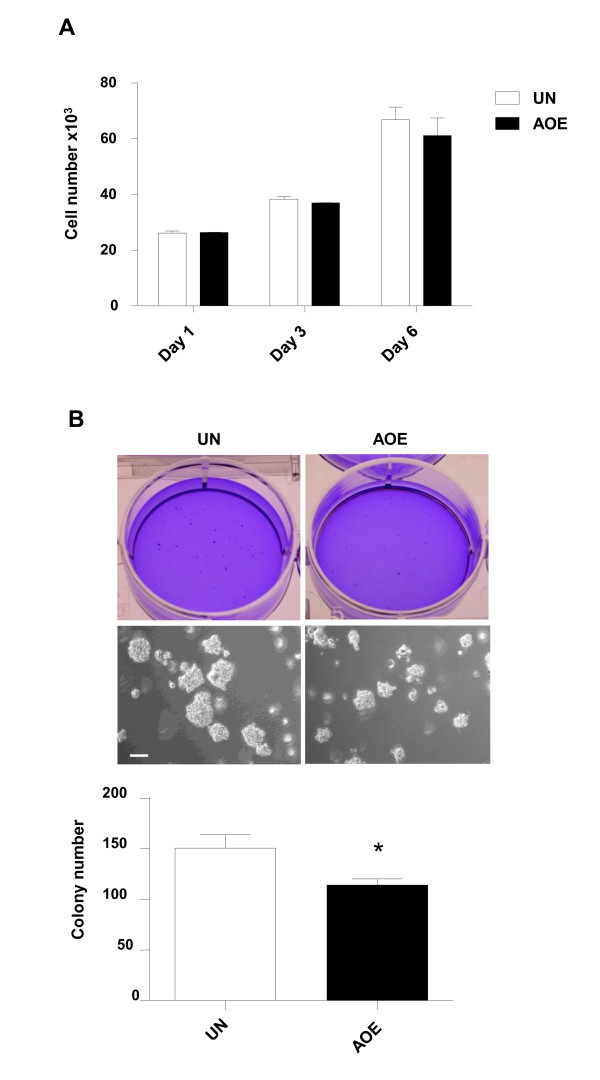
**Effect of epigenetic reprogramming on malignant cell phenotype**. Cancer cell growth after reprogramming with AOE. **(A) **Proliferation of MCF-7 cells after 1, 3 and 6 days of culture in adherent conditions as measured by MTT assay. **(B) **Growth of MCF-7 cells in anchorage-independent conditions. The top panels show cultures stained with crystal violet and representative fields of view of the same cultures in soft agar. The bottom panel show quantification of colony number as counted under a stereomicrosope. Bar = 100 μm. * indicates P < 0.05.

In this study we show that *RARB*, *CST6*, *CCND2*, *GAS2 *and *CDKN2A *tumour suppressor genes are reprogrammed by oocyte extracts. Because these genes control cell growth, death and invasion, we next studied the effect of reversing the silenced expression state on the malignancy of breast cancer cells. Again, AOE was used because of its superior reprogramming activity. Figure 5A shows no difference in cell numbers when cancer cells were maintained in culture for 1, 3, or 6 days after reprogramming by AOE, indicating that extract treatment is non-toxic, and does not affect cell proliferation after permeabilisation. We could not detect any difference in the distribution of cells through G1, S, or G2/M phases of the cell cycle, nor in the percentage of apoptotic cells, when analysis was done at the same time points (Additional file 4: Figure S4). We next tested whether reprogramming affected the ability of transformed MCF-7 cells to grow in anchorage-independent conditions by performing soft agar assay. After 2 weeks of growth in agar, control or treated cells formed colonies that were counted under a stereomicroscope. A significant reduction of colony size and number was observed for cells treated with AOE (Figure 5B). The same effect was not obtained when non-permeabilised cells were exposed to AOE (Additional file 4: Figure S5). Reduction of cancer cell proliferation, and induction of apoptosis, has been reported in previous tumour reversion experiments. For example, exposure to zebrafish embryo extracts can reduce two dimensional growth of colon cancer cells [39]. Similar effects have been reported for breast cancer cells grown in soft agar after exposure to Matrigel conditioned with human embryonic stem cells [13], or in response to co-culture with human umbilical cord matrix stem cells [40]. These effects are mediated by extracellular factors, such as Nodal and cytokines; however, we observed an effect of AOE on cancer cell proliferation only after permeabilisation, suggesting that reprogramming mediated by oocyte extract molecules is not signalled through receptors on the cell surface, but rather by the direct association of oocyte molecules with the chromatin of cancer cells.

**Figure 6:**
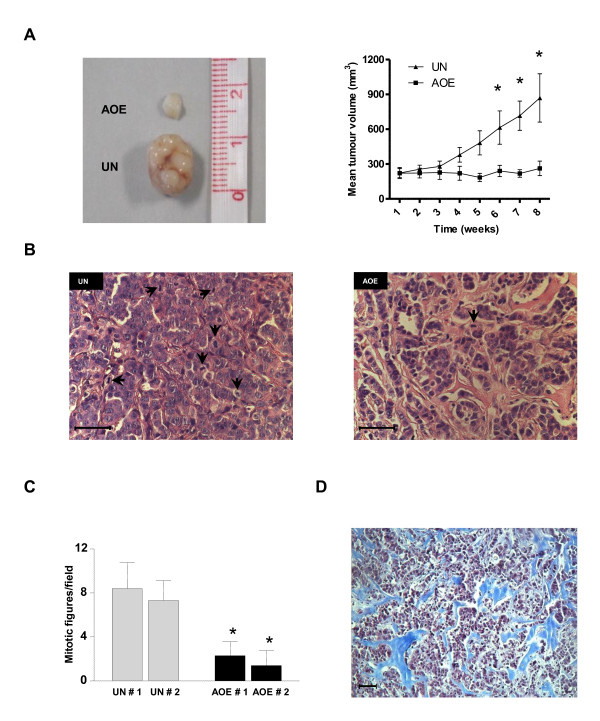
**Effect of epigenetic reprogramming on *in vivo *tumorigenicity**. Macroscopic and microscopic analyses of tumour xenografts. **(A) **Macroscopic appearance of untreated (UN) and AOE-treated tumour xenografts (AOE) and relative tumour growth curves (* indicates P < 0.05). **(B) **Eosin-Haematoxylin staining of untreated tumour sections (black arrows: mitotic figures; Bar = 50 μm). **(C) **Number of mitotic figures for two independent tumours in untreated (UN) and AOE-treated xenografts (AOE) (* indicates P < 0.05). **(D) **Interstitial stroma present in AOE-treated tumours stained for collagen (blue staining; Bar = 50 μm).

We next investigated the effect of AOE on cancer cell growth *in vivo *after transplantation into immuno-compromised mice. Tumours from reprogrammed cells were significantly smaller than those from untreated cells when analyzed after 8 weeks of transplantation (Figure 6A). Histologically, both tumours appeared well circumscribed but not encapsulated, with high degree of nuclear pleomorphism. In addition, AOE-treated tumours showed a significant decrease in the number of mitotic divisions (Figure 6B: black arrows; Figure 6C). The reduced tumour growth was associated with a reduction of epithelial cells and an increase in interstitial stroma (Figure 6C), which stained positive for collagen (Figure 6D). These results show that the epigenetic reprogramming induced by AOE induces stable changes that result in long term suppression of tumorigenicity. It will be now important to identify the oocyte-specific molecules involved in this process, and the molecular pathways responsible for the arrest of tumour growth. In our view, the identification of these molecules will provide a rich source of information for the design of synthetic molecules that can be used for pharmaceutical interventions.

## Conclusions

This study describes a new method for investigating reprogramming of silenced tumour suppressor genes using oocyte extracts. Understanding the molecular mechanisms of tumourigenesis has been central to cancer research for decades and reports of tumour reversion are creating exciting avenues to address this important biological and biomedical question. Reprogramming in response to embryonic environments [8] or by over-expression of embryonic factors [41,42] can change cancer cell fate over many cell divisions. Reprogramming with oocyte molecules which does not require DNA replication, can directly remodel cancer cell chromatin and induce re-expression of silenced tumour suppressor genes after only 6 hours of treatment. The long-term effects of this treatment are reflected in reduced tumour growth *in vivo*. This reprogramming system can easily be adapted to understand how oocyte chromatin remodelers and DNA demethylating complexes are targeted to promoter regions of tumour suppressor genes. Since oocyte-mediated demethylation does not occur randomly, this system can be employed to identify the mechanisms that maintain tumour suppressor gene silencing in cancer cells. Although this study focussed on reprogramming of selected tumour suppressor genes, we show that AOE can induce stable epigenetic reprogramming. Future studies will focus on pure populations of reprogrammed cells, isolated by selection in culture, to study their epigenotype at a genome-wide level. In this study we have used cancer cell lines with complex genetic abnormalities. Since NT studies highlight that genetically abnormal cells are resistant to tumour reversion [16], future studies will test oocyte-mediated reprogramming on cells derived from early stage tumours to elucidate the contribution of epigenetic alterations to the onset of breast oncogenesis.

Our data show that DNA methylation represents a bottleneck to reprogramming since extracts of ESC cannot efficiently reprogram hypermethylated tumour suppressor genes. Defining the relationship between different levels of epigenetic regulation for cancer-related genes is essential for devising epigenetic therapies and this system could be of paramount importance for dissecting this aspect of the problem. Extracts of axolotl oocytes show superior reprogramming capacity and they present several practical advantages, including cell size and availability. We propose that they can be a valuable tool to understand how cells become malignant and to advance the discovery of novel cancer therapies.

## Methods

### Cell culture and permeabilization

All culture reagents were from Invitrogen and chemicals from Sigma unless otherwise indicated. Cells were incubated in a 37°C humidified incubator with 5% C0_2_.

MCF-7 and HCC1954 cell lines were purchased from ATCC and maintained in RPMI medium containing 10% fetal calf serum (FCS), 2 mM glutamine, 1% non-essential amino acids, 1% sodium pyruvate, and 1% penicillin/streptomycin. HMEC cells and HMEC complete medium were from Invitrogen. HMEC were passaged by incubation with 0.25% trypsin for 15 min at 37°C and neutralisation with 0.1% soybean trypsin inhibitor. CGR8 mouse ESC were obtained from ECACC and cultured on gelatin-coated culture dishes in DMEM containing 15% Hyclone stem cell screened FCS, 2 mM glutamine, 1% non-essential amino acids, 1% sodium pyruvate, 1% penicillin/streptomycin, 1000 U/ml of leukemia inhibitory factor (Millipore) and 0.1 mM beta-mercaptoethanol. NTERA2 cells (kindly donated by Prof. Andrews, University of Sheffield) were cultured in DMEM medium containing 10% FCS, 2 mM glutamine, 1% non-essential amino acids, and 1% penicillin/streptomycin.

Cell permeabilisation was performed as previously reported [21]. Briefly, cell suspensions (2 × 10^6 ^cells/ml) were treated with 20 μg/ml digitonin in PB buffer (170 mM potassium gluconate, 5 mM KCl, 2 mM MgCl_2_, 1 mM KH_2_PO_4_, 1 mM EGTA, 20 mM Hepes, supplemented with 3 μg/ml leupeptin, 1 μg/ml aprotinin and 1 μg/ml pepstatin A, pH 7.25, freshly prepared prior to use) for 1-2 min on ice. Cells were washed in cold PB buffer and permeabilisation was assessed by staining with propidium iodide (PI) and 70 kDa FITC-dextran.

### Treatment of cells in oocyte, egg and ESC extracts

Axolotl and *Xenopus *oocyte/egg extracts (AOE, XOE and XEE, respectively) were prepared from mature females as described previously [21,23]. Mouse ESC extracts (ESCE) were prepared according to Taranger et al., [31].

Permeabilised cells were added to oocyte/egg and ESC extracts (5,000 cells/μl extract) supplemented with an energy regenerating system (150 μg/ml creatine phosphokinase, 60 mM phosphocreatine, 1 mM ATP) and incubated at 17°C for AOE, 21°C for XOE and XEE, and 37°C for ESCE.

### Gene expression analysis

Cells were collected and processed for RNA extraction using Qiagen RNAeasy mini kit with Qiashredder and DNAse treatment. cDNA synthesis was performed with Superscript III reverse transcriptase (Invitrogen). Real time PCR (Q-PCR) was performed using the 7500 Fast Real-Time PCR System (Applied Biosystems). TaqMan Gene expression master mix and TaqMan Gene expression assays were used (assay ID can be found in Additional file 5: Table S1). After validation of the amplification efficiencies, the Relative Quantification method (ΔΔCt) was used to quantify the gene expression levels of each gene relative to *ACTB *(*ACTIN*, endogenous control) for each sample. Results are represented as fold increase in expression relative to untreated sample (UN) used as calibrator (mean ± sd, n = 3).

### Western Blotting

Nuclear proteins were extracted with NucBuster™ Protein Extraction Kit (Calbiochem).

Extracted proteins were loaded into a 12% Acrylamide gel (10 μg/lane), separated by SDS-PAGE electrophoresis and blotted onto a PVDF membrane. Membranes were blocked with 10% skimmed milk and then probed overnight at 4°C with a rabbit anti-NANOG antibody (1:1,000, Peprotech) or goat anti-OCT-4 antibody (1:1,000, Santa Cruz Biotechnology), in the presence of 0.05% Tween 20 and 5% milk. Peroxidase conjugated donkey anti-rabbit (1:10,000; GE Healthcare) or anti-goat (1:10,000; Sigma) antibodies were incubated for 1h at RT. ECL plus kit (Amersham Biosciences) was used to detect chemiluminescence.

### Bisulfite genomic sequencing

Genomic DNA was isolated using DNeasy Tissue kit (Qiagen). Bisulfite genomic sequencing was carried out as described previously [43]. Briefly, 1 μg of genomic DNA was used for bisulfite treatment (5 hours, 55°C) and 1 μl of bisulfite converted DNA was used for PCR reactions using 2.5 U of Platinum Taq polymerase (Invitrogen) (primers are listed in Additional file 5: Table S1). Primers spanning CpG island sequences were designed using Methprimer software http://www.urogene.org/methprimer/index1.html. Purified PCR products were either directly sequenced or cloned into pGEM-T easy (Promega), with 10 or more clones of each sample subjected to sequencing.

### Chromatin Immunoprecipitation (ChIP)

ChIP experiments were performed using the Magna ChIP A kit (Millipore). One million cells were used with the following antibodies: ChIP grade rabbit polyclonal anti-H3K27me3 (3 μg, Millipore 07-449), ChIP grade rabbit polyclonal anti-H3K4me3 (3 μg, Abcam ab8580), ChIP grade rabbit polyclonal anti-H3K9ac (1.5 μg, Abcam ab4441), ChIP grade rabbit polyclonal anti-H3K9me3 (2 μg, Abcam ab8898), ChIP grade mouse monoclonal anti-H3K9me2 (2 μg, Abcam ab1220), IgG from rabbit serum (4 μg, Sigma I5006). Immunoprecipitated DNA was quantified by Q-PCR using the 7500 Fast Real-Time PCR System (Applied Biosystems) with 5 μl DNA (from a total of 50 μl). TaqMan Gene expression master mix and TaqMan Gene expression custom assays (Applied Biosystem) were used (primers and probes are listed in Additional file 5: Table S1). Data are presented as "Fold enrichment" of precipitated DNA for each histone modification relative to a 1/100 dilution of input chromatin (mean ± sd, n = 3).

### Luciferase assay

HMEC, MCF-7 and HC1954 cells were transfected with Firefly *RARB *reporter and Renilla luciferase transfection control *pRL-TK *(Promega) plasmids using Lipofectamine 2000 (Invitrogen). The *RARB *reporter (containing promoter sequence fragment -522 to +156) was obtained by cloning into pGL3-Basic (Promega) at the Nhe and Xho1 restriction sites in both orientations. The vector with antisense promoter orientation was used as control in transfection experiments. Retinoic acid (RA 1 μM, Sigma) treatment was performed for 24 hours. After normalizing the Firefly values to Renilla, the data are presented as relative luciferase values to the negative control (mean ± sd, n = 3).

### Cell proliferation assay

Permeabilised MCF-7 cells were incubated in AOE and after 6 hours plated at a density of 12.5 × 10^3 ^cells/cm^2 ^in triplicate. After 1, 3, and 6 days in culture MTT was added at 5 mg/ml and incubated for 3 hours at 37°C. The converted dye was dissolved by treatment with isopropanol containing 0.04N HCl and quantified by measuring the absorbance at 570 nm with background subtraction at 650 nm in a SmartSpec 3000 Spectrophotometer (Bio-Rad Laboratories). Results are presented as mean ± sd, n = 3.

### Apoptosis and cell cycle assays

MCF-7 cells treated in AOE were plated at a density of 5 × 10^3 ^cells/cm^2 ^in triplicate. After 1, 3, and 6 days in culture cells were trypsinised and fixed with 70% ice-cold ethanol for 30 min at -20°C. Cell were then centrifuged and stained in 50 μg/ml PI solution containing 0.1 mg/ml RNase A and 0.05% Triton X-100 for 30 min. After washing, cells were resuspended in PBS and 50,000 cells analysed with a Beckman Coulter FC-500 flow cytometer.

### Soft agar assay

MCF-7 cells (30,000/6 well) were seeded in 0.5 ml of 0.3% noble agar in complete RPMI medium overlaying a 1 ml 0.5% agar in the same medium. After 2 weeks culture cell colonies were stained with crystal violet and colonies ≥ of 100 μm counted under a MZ12_5 _Leica stereomicroscope. For control experiments with non-permeabilised cells, cells were either incubated with AOE for 6 hours and then plated in soft agar or cultured with different amounts of AOE into the agar top layer. In the latter case 10, 50 and 100 μl of AOE were added to the top layer of soft agar together with MCF-7 cells (corresponding to the same, 5-fold and 10-fold higher ratio of cell/extract used with permeabilised cells).

### Tumour xenografts

Female MF1 nude mice (Harlan-Olac) were anaesthetised with Ketamine/Medetomidine and MCF-7 cells at 1.5 × 10^6 ^cells in a volume of 200 μl Matrigel were injected sub-cutaneously into the left flank. In addition, 0.1 mg 17-beta-estradiol pellet (60-day release; Innovative Research of America, US) implanted subcutaneously into the scruff of each mouse. Tumour dimensions were measured by calliper measurement of length and width three times weekly and the volume calculated [(length^2 ^× width)/2] and clinical condition of the mice were monitored by weekly body weight measurements for the duration of the study (n = 4-6 for each time point). The project was run under Home Office project PPL 40/2962 which was awarded in November 2006 (Watson) following local ethical approval. The study also adhered to the UK Co-ordinating Committee for Cancer Research (UKCCCR) guidelines. At termination, tumours were excised, fixed in formalin and paraffin embedded.

### Histochemistry

Histology sections (5 μm) were stained with Eosin & Haematoxylin and observed under a Leica DM5000B microscope and Leica Application Suite software.

Mitotic figures were quantified by examination of 10 fields of view at high power magnification (630x) in two independent tumours. Collagen was stained with the Trichrome Stains (Masson, Sigma) according to manufacturer's instructions.

### Statistical analysis

GraphPad InStat3 software was used to perform statistical analysis. Q-PCR and ChIP data were analysed by one-way ANOVA with post Tukey's multiple comparison test with a significance level set at P < 0.05. Bisulfite sequencing data were analysed by χ^2 ^test with a significance level set at P < 0.05. Cell proliferation, soft agar assay and luciferase assay, data were analysed with unpaired Student's t-test (P < 0.05). Tumour growth data were analysed by two-way ANOVA with Bonferroni post-test (P < 0.05). Mitotic figures data were analysed by one-way ANOVA with post Tukey's multiple comparison test with a significance level set at P < 0.05.

## Competing interests

This work was funded by Evocell Ltd. RA and ADJ are share holders of Evocell Ltd.

## Authors' contributions

CA conceived the study and performed gene expression assays, bisulfite sequencing, ChIP, cell proliferation, apoptosis and soft agar assays. MDR contributed with gene expression assays and bisulfite sequencing. JD made the *RARB *reporter plasmid. RK and SW performed mouse xenograft experiments. MS and VS contributed to transplantation experiments. CA, RA, ADJ planned the experiments, discussed the results and wrote the paper. All authors read and approved the final manuscript.

## Supplementary Material

Additional file 1 **Permeabilisation and viability of reprogrammed cancer cells**. The **Figure S1 **shows permeabilisation and viability of MCF-7 cells after permeabilisation with digitonin and incubation in AOE. **(A) **FITC-dextran (green) staining of the cytoplasm of permeabilised cells. Note exclusion of dextran from the nucleus. **(B) **PI (red) staining of the nucleus of permabilised cells. **(C) **Digitonin-treated cells show both cellular and nuclear membrane permeability with preservation of cytoplasm (merge). **(D) **Permeabilised cells treated with AOE for 6 hours are viable and show presence of vacuoles due to treatment with digitonin after 3 days in culture. Click here for file

Additional file 2 **Effect of AOE-mediated reprogramming on expression of pluripotency genes**. The **Figure S2 **shows methylation of *OCT-4 *and *NANOG *promoters and relative protein expression after reprogramming with AOE. **(A) **Methylation of *OCT-4 *and *NANOG *promoters by direct sequencing after bisulfite conversion of DNA. Schematics indicate the position of analysed CpG islands in promoter regions. Black circles indicate metylated CGs, black/white circles indicate partially methylated CGs. **(B) **Expression of OCT-4 and NANOG protein by Western Blotting (10 μg protein/lane). NTERA2 cells were used as positive control for expression of pluripotency genes. The Coomassie stained SDS-PAGE gel is shown as loading control. Click here for file

Additional file 3 **Reprogramming of *GAS2 *histone marks by ESCE**. Analysis of *GAS2 *gene promoter by ChIP. Data are presented as fold enrichment to input chromatin and indicate reprogramming of histone repressive (H3K9me3, H3K9me2, H3K27me3) and active (H3K4me3, H3K9Ac) marks by ESCE after 6 hours of treatment. * indicates P < 0.05 for treated groups different from UN. Click here for file

Additional file 4 **Effect of reprogramming with AOE on cancer cell growth**. The data provided show the effect of epigenetic reprogramming by AOE on MCF-7 cells. **Figure S4: Cell cycle analysis of reprogrammed cells**. Cell cycle profiles of control and AOE-treated cells analysed after 1, 3 and 6 days of treatment. **Figure S5: Effect of AOE on growth of non-permeabilised cells in soft agar**. Representative images of soft agar assay where different quantity of AOE (10, 50 or 100 μl AOE: equivalent to the same, 5-fold and 10-fold the quantity of extract per number of cells used in experiments with permeabilisation) were included in the top agar layer with MCF-7 cells. Equivalent results were obtained when non-permeabilised cells were incubated with AOE for 6 hours and cultured in soft agar. Bar = 100 μm. Click here for file

Additional file 5 **Q-PCR assay ID, primers and probes**. The **Table S1 **lists the TaqMan gene expression assays, primers and probes used in this study. Click here for file

## References

[B1] Sadikovic B Al-Romaih K Squire JA Zielenska M Cause and consequences of genetic and epigenetic alterations in human cancer Curr Genomics 2008 9 394 408 10.2174/138920208785699580 19506729 PMC2691666

[B2] Jones PA Laird PW Cancer epigenetics comes of age Nat Genet 1999 21 163 167 10.1038/5947 9988266

[B3] Lo PK Sukumar S Epigenomics and breast cancer Pharmacogenomics 2008 9 1879 1902 10.2217/14622416.9.12.1879 19072646 PMC2633440

[B4] Novak P Jensen TJ Garbe JC Stampfer MR Futscher BW Stepwise DNA methylation changes are linked to escape from defined proliferation barriers and mammary epithelial cell immortalization Cancer Res 2009 69 5251 5258 10.1158/0008-5472.CAN-08-4977 19509227 PMC2697259

[B5] Hinshelwood RA Clark SJ Breast cancer epigenetics: normal human mammary epithelial cells as a model system J Mol Med 2008 86 1315 1328 10.1007/s00109-008-0386-3 18716754

[B6] Tommasi S Karm DL Wu X Yen Y Pfeifer GP Methylation of homeobox genes is a frequent and early epigenetic event in breast cancer Breast Cancer Res 2009 11 R14 10.1186/bcr2233 19250546 PMC2687719

[B7] Telerman A Amson R The molecular programme of tumour reversion: the steps beyond malignant transformation Nat Rev Cancer 2009 9 206 216 10.1038/nrc2589 19180095

[B8] Hendrix MJ Seftor EA Seftor RE Kasemeier-Kulesa J Kulesa PM Postovit LM Reprogramming metastatic tumour cells with embryonic microenvironments Nat Rev Cancer 2007 7 246 255 10.1038/nrc2108 17384580

[B9] Mintz B Illmensee K Normal genetically mosaic mice produced from malignant teratocarcinoma cells Proc Natl Acad Sci USA 1975 72 3585 3589 10.1073/pnas.72.9.3585 1059147 PMC433040

[B10] Lee LM Seftor EA Bonde G Cornell RA Hendrix MJ The fate of human malignant melanoma cells transplanted into zebrafish embryos: assessment of migration and cell division in the absence of tumor formation Dev Dyn 2005 233 1560 1570 10.1002/dvdy.20471 15968639

[B11] Kulesa PM Kasemeier-Kulesa JC Teddy JM Margaryan NV Seftor EA Seftor RE Hendrix MJ Reprogramming metastatic melanoma cells to assume a neural crest cell-like phenotype in an embryonic microenvironment Proc Natl Acad Sci USA 2006 103 3752 3757 10.1073/pnas.0506977103 16505384 PMC1450149

[B12] Diez-Torre A Andrade R Eguizabal C Lopez E Arluzea J Silio M Arechaga J Reprogramming of melanoma cells by embryonic microenvironments Int J Dev Biol 2009 53 1563 1568 10.1387/ijdb.093021ad 19924629

[B13] Postovit LM Margaryan NV Seftor EA Kirschmann DA Lipavsky A Wheaton WW Abbott DE Seftor RE Hendrix MJ Human embryonic stem cell microenvironment suppresses the tumorigenic phenotype of aggressive cancer cells Proc Natl Acad Sci USA 2008 105 4329 4334 10.1073/pnas.0800467105 18334633 PMC2393795

[B14] Wilmut I Schnieke AE McWhir J Kind AJ Campbell KH Viable offspring derived from fetal and adult mammalian cells Nature 1997 385 810 813 10.1038/385810a0 9039911

[B15] Li L Connelly MC Wetmore C Curran T Morgan JI Mouse embryos cloned from brain tumors Cancer Res 2003 63 2733 2736 12782575

[B16] Blelloch RH Hochedlinger K Yamada Y Brennan C Kim M Mintz B Chin L Jaenisch R Nuclear cloning of embryonal carcinoma cells Proc Natl Acad Sci USA 2004 101 13985 13990 15306687 10.1073/pnas.0405015101 PMC521109

[B17] Hochedlinger K Blelloch R Brennan C Yamada Y Kim M Chin L Jaenisch R Reprogramming of a melanoma genome by nuclear transplantation Genes Dev 2004 18 1875 1885 10.1101/gad.1213504 15289459 PMC517407

[B18] Alberio R Campbell KH Johnson AD Reprogramming somatic cells into stem cells Reproduction 2006 132 709 720 10.1530/rep.1.01077 17071772

[B19] Bui HT Wakayama S Kishigami S Kim JH Van Thuan N Wakayama T The cytoplasm of mouse germinal vesicle stage oocytes can enhance somatic cell nuclear reprogramming Development 2008 135 3935 3945 10.1242/dev.023747 18997114

[B20] Miyamoto K Tsukiyama T Yang Y Li N Minami N Yamada M Imai H Cell-free extracts from mammalian oocytes partially induce nuclear reprogramming in somatic cells Biol Reprod 2009 80 935 943 10.1095/biolreprod.108.073676 19164171

[B21] Alberio R Johnson AD Stick R Campbell KH Differential nuclear remodeling of mammalian somatic cells by Xenopus laevis oocyte and egg cytoplasm Exp Cell Res 2005 307 131 141 10.1016/j.yexcr.2005.02.028 15922733

[B22] Simonsson S Gurdon J DNA demethylation is necessary for the epigenetic reprogramming of somatic cell nuclei Nat Cell Biol 2004 6 984 990 10.1038/ncb1176 15448701

[B23] Bian Y Alberio R Allegrucci C Campbell KH Johnson AD Epigenetic marks in somatic chromatin are remodelled to resemble pluripotent nuclei by amphibian oocyte extracts Epigenetics 2009 4 194 202 10.4161/epi.4.3.8787 19440040

[B24] Bachvarova RF Crother BI Johnson AD Evolution of germ cell development in tetrapods: comparison of urodeles and amniotes Evol Dev 2009 11 603 609 10.1111/j.1525-142X.2009.00366.x 19754716

[B25] Johnson AD Crother B White ME Patient R Bachvarova RF Drum M Masi T Regulative germ cell specification in axolotl embryos: a primitive trait conserved in the mammalian lineage Philos Trans R Soc Lond B Biol Sci 2003 358 1371 1379 10.1098/rstb.2003.1331 14511484 PMC1693234

[B26] Johnson AD Drum M Bachvarova RF Masi T White ME Crother BI Evolution of predetermined germ cells in vertebrate embryos: implications for macroevolution Evol Dev 2003 5 414 431 10.1046/j.1525-142X.2003.03048.x 12823457

[B27] Swiers G Chen YH Johnson AD Loose M A conserved mechanism for vertebrate mesoderm specification in urodele amphibians and mammals Dev Biol 2010 343 138 152 10.1016/j.ydbio.2010.04.002 20394741

[B28] Dixon JE Allegrucci C Redwood C Kump K Bian Y Chatfield J Chen YH Sottile V Voss SR Alberio R Johnson AD Axolotl Nanog activity in mouse embryonic stem cells demonstrates that ground state pluripotency is conserved from urodele amphibians to mammals Development 2010 137 2973 80 10.1242/dev.049262 20736286 PMC2926951

[B29] Clark SJ Action at a distance: epigenetic silencing of large chromosomal regions in carcinogenesis Hum Mol Genet 2007 16 R88 95 10.1093/hmg/ddm051 17613553

[B30] Bru T Clarke C McGrew MJ Sang HM Wilmut I Blow JJ Rapid induction of pluripotency genes after exposure of human somatic cells to mouse ES cell extracts Exp Cell Res 2008 314 2634 2642 10.1016/j.yexcr.2008.05.009 18571647 PMC2577761

[B31] Taranger CK Noer A Sorensen AL Hakelien AM Boquest AC Collas P Induction of dedifferentiation, genomewide transcriptional programming, and epigenetic reprogramming by extracts of carcinoma and embryonic stem cells Mol Biol Cell 2005 16 5719 5735 10.1091/mbc.E05-06-0572 16195347 PMC1289416

[B32] Hajkova P Jeffries SJ Lee C Miller N Jackson SP Surani MA Genome-wide reprogramming in the mouse germ line entails the base excision repair pathway Science 2010 329 78 82 10.1126/science.1187945 20595612 PMC3863715

[B33] Brandeis M Frank D Keshet I Siegfried Z Mendelsohn M Nemes A Temper V Razin A Cedar H Sp1 elements protect a CpG island from de novo methylation Nature 1994 371 435 438 10.1038/371435a0 8090226

[B34] Wierstra I Sp1: emerging roles--beyond constitutive activation of TATA-less housekeeping genes Biochem Biophys Res Commun 2008 372 1 13 10.1016/j.bbrc.2008.03.074 18364237

[B35] Jeter CR Badeaux M Choy G Chandra D Patrawala L Liu C Calhoun-Davis T Zaehres H Daley GQ Tang DG Functional evidence that the self-renewal gene NANOG regulates human tumor development Stem Cells 2009 27 993 1005 10.1002/stem.29 19415763 PMC3327393

[B36] Kondo Y Shen L Cheng AS Ahmed S Boumber Y Charo C Yamochi T Urano T Furukawa K Kwabi-Addo B Gold DL Sekido Y Hang TH Issa IP Gene silencing in cancer by histone H3 lysine 27 trimethylation independent of promoter DNA methylation Nat Genet 2008 40 741 750 10.1038/ng.159 18488029

[B37] McGarvey KM Fahrner JA Greene E Martens J Jenuwein T Baylin SB Silenced tumor suppressor genes reactivated by DNA demethylation do not return to a fully euchromatic chromatin state Cancer Res 2006 66 3541 3549 10.1158/0008-5472.CAN-05-2481 16585178

[B38] Hansis C Barreto G Maltry N Niehrs C Nuclear reprogramming of human somatic cells by xenopus egg extract requires BRG1 Curr Biol 2004 14 1475 1480 10.1016/j.cub.2004.08.031 15324664

[B39] Cucina A Biava PM D'Anselmi F Coluccia P Conti F di Clemente R Miccheli A Frati L Gulino A Bizzarri M Zebrafish embryo proteins induce apoptosis in human colon cancer cells (Caco2) Apoptosis 2006 11 1617 1628 10.1007/s10495-006-8895-4 16820966

[B40] Ayuzawa R Doi C Rachakatla RS Pyle MM Maurya DK Troyer D Tamura M Naive human umbilical cord matrix derived stem cells significantly attenuate growth of human breast cancer cells in vitro and in vivo Cancer Lett 2009 280 31 37 10.1016/j.canlet.2009.02.011 19285791 PMC2914472

[B41] Miyoshi N Ishii H Nagai KI Hoshino H Mimori K Tanaka F Nagano H Sekimoto M Doki Y Mori M Defined factors induce reprogramming of gastrointestinal cancer cells Proc Natl Acad Sci USA 2010 107 40 45 10.1073/pnas.0912407107 20018687 PMC2806714

[B42] Utikal J Maherali N Kulalert W Hochedlinger K Sox2 is dispensable for the reprogramming of melanocytes and melanoma cells into induced pluripotent stem cells J Cell Sci 2009 122 3502 3510 10.1242/jcs.054783 19723802 PMC2746132

[B43] Allegrucci C Wu YZ Thurston A Denning CN Priddle H Mummery CL Ward-van Oostwaard D Andrews PW Stojkovic M Smith N Parkin T Jones ME Warren G Yu L Brena RM Plass C Young LE Restriction landmark genome scanning identifies culture-induced DNA methylation instability in the human embryonic stem cell epigenome Hum Mol Genet 2007 16 1253 1268 10.1093/hmg/ddm074 17409196

