# Supplementary figures and images for: Epigenetic reprogramming of breast cancer cells with oocyte extracts
Source: Mol Cancer. 2011 Jan 13;10:7. doi: 10.1186/1476-4598-10-7 (PMC3034708; doi:10.1186/1476-4598-10-7)

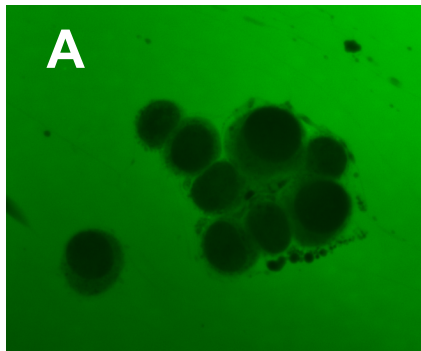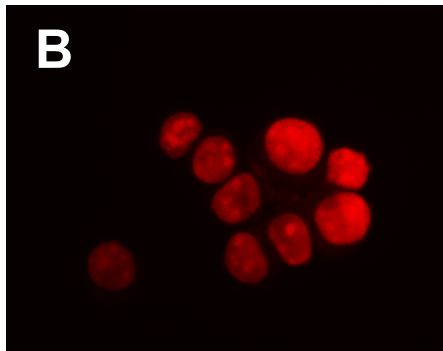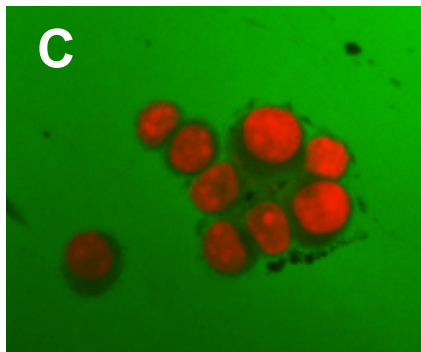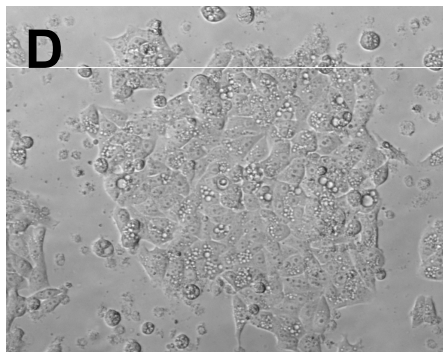

Supplement: Additional file 1 — Permeabilisation and viability of reprogrammed cancer cells. The Figure S1 shows permeabilisation and viability of MCF-7 cells after permeabilisation with digitonin and incubation in AOE. (A) FITC-dextran (green) staining of the cytoplasm of permeabilised cells. Note exclusion of dextran from the nucleus. (B) PI (red) staining of the nucleus of permabilised cells. (C) Digitonin-treated cells show both cellular and nuclear membrane permeability with preservation of cytoplasm (merge). (D) Permeabilised cells treated with AOE for 6 hours are viable and show presence of vacuoles due to treatment with digitonin after 3 days in culture. [file 1476-4598-10-7-S1.PDF]

A

*OCT-4*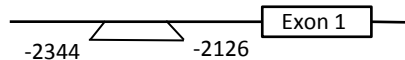

6 hours

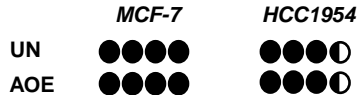

6 days

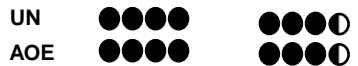*NANOG*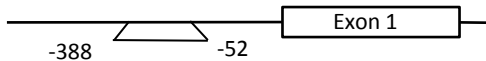

6 hours

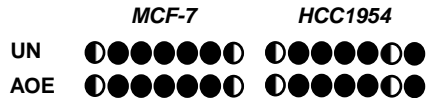

6 days

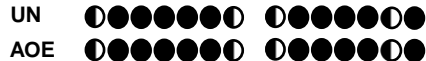

B

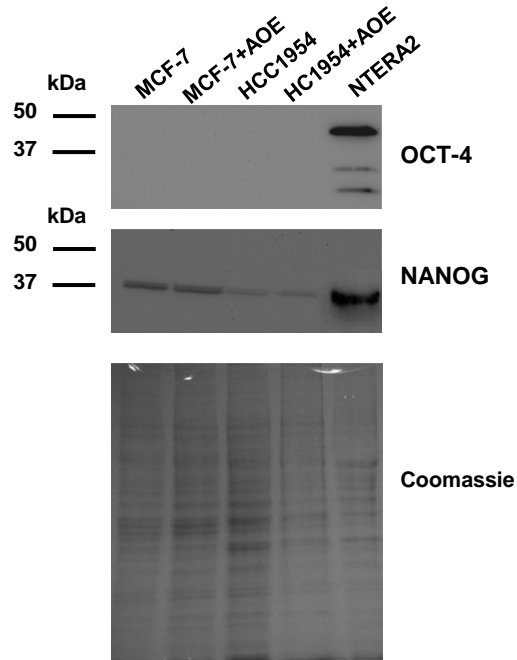

Supplement: Additional file 2 — Effect of AOE-mediated reprogramming on expression of pluripotency genes. The Figure S2 shows methylation of OCT-4 and NANOG promoters and relative protein expression after reprogramming with AOE. (A) Methylation of OCT-4 and NANOG promoters by direct sequencing after bisulfite conversion of DNA. Schematics indicate the position of analysed CpG islands in promoter regions. Black circles indicate metylated CGs, black/white circles indicate partially methylated CGs. (B) Expression of OCT-4 and NANOG protein by Western Blotting (10 μg protein/lane). NTERA2 cells were used as positive control for expression of pluripotency genes. The Coomassie stained SDS-PAGE gel is shown as loading control. [file 1476-4598-10-7-S2.PDF]

## *GAS2*

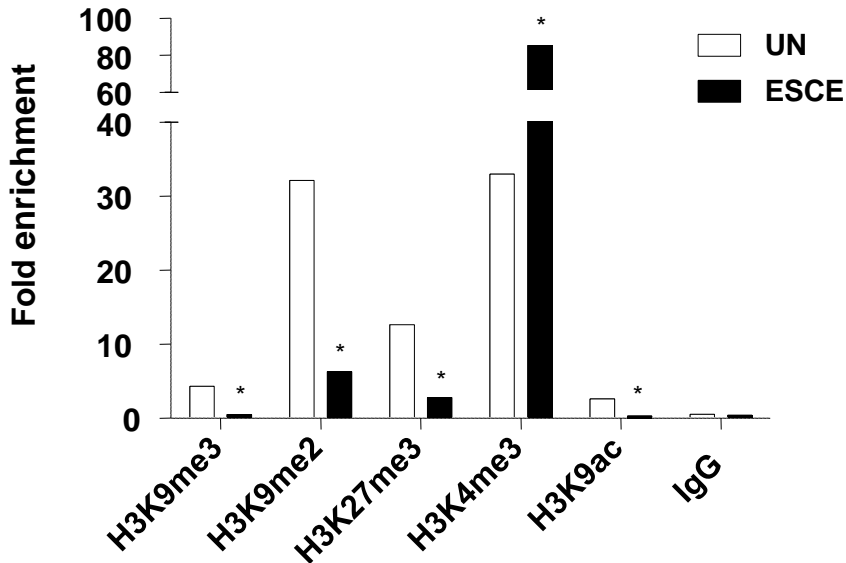

Supplement: Additional file 3 — Reprogramming of GAS2 histone marks by ESCE. Analysis of GAS2 gene promoter by ChIP. Data are presented as fold enrichment to input chromatin and indicate reprogramming of histone repressive (H3K9me3, H3K9me2, H3K27me3) and active (H3K4me3, H3K9Ac) marks by ESCE after 6 hours of treatment. * indicates P < 0.05 for treated groups different from UN. [file 1476-4598-10-7-S3.PDF]
