# Supplementary material for: Epigenetic reprogramming of breast cancer cells with oocyte extracts
Source: Mol Cancer. 2011 Jan 13;10:7. doi: 10.1186/1476-4598-10-7 (PMC3034708; doi:10.1186/1476-4598-10-7)
Supplement: Additional file 4 — Effect of reprogramming with AOE on cancer cell growth. The data provided show the effect of epigenetic reprogramming by AOE on MCF-7 cells. Figure S4: Cell cycle analysis of reprogrammed cells. Cell cycle profiles of control and AOE-treated cells analysed after 1, 3 and 6 days of treatment. Figure S5: Effect of AOE on growth of non-permeabilised cells in soft agar. Representative images of soft agar assay where different quantity of AOE (10, 50 or 100 μl AOE: equivalent to the same, 5-fold and 10-fold the quantity of extract per number of cells used in experiments with permeabilisation) were included in the top agar layer with MCF-7 cells. Equivalent results were obtained when non-permeabilised cells were incubated with AOE for 6 hours and cultured in soft agar. Bar = 100 μm. [file 1476-4598-10-7-S4.PDF]

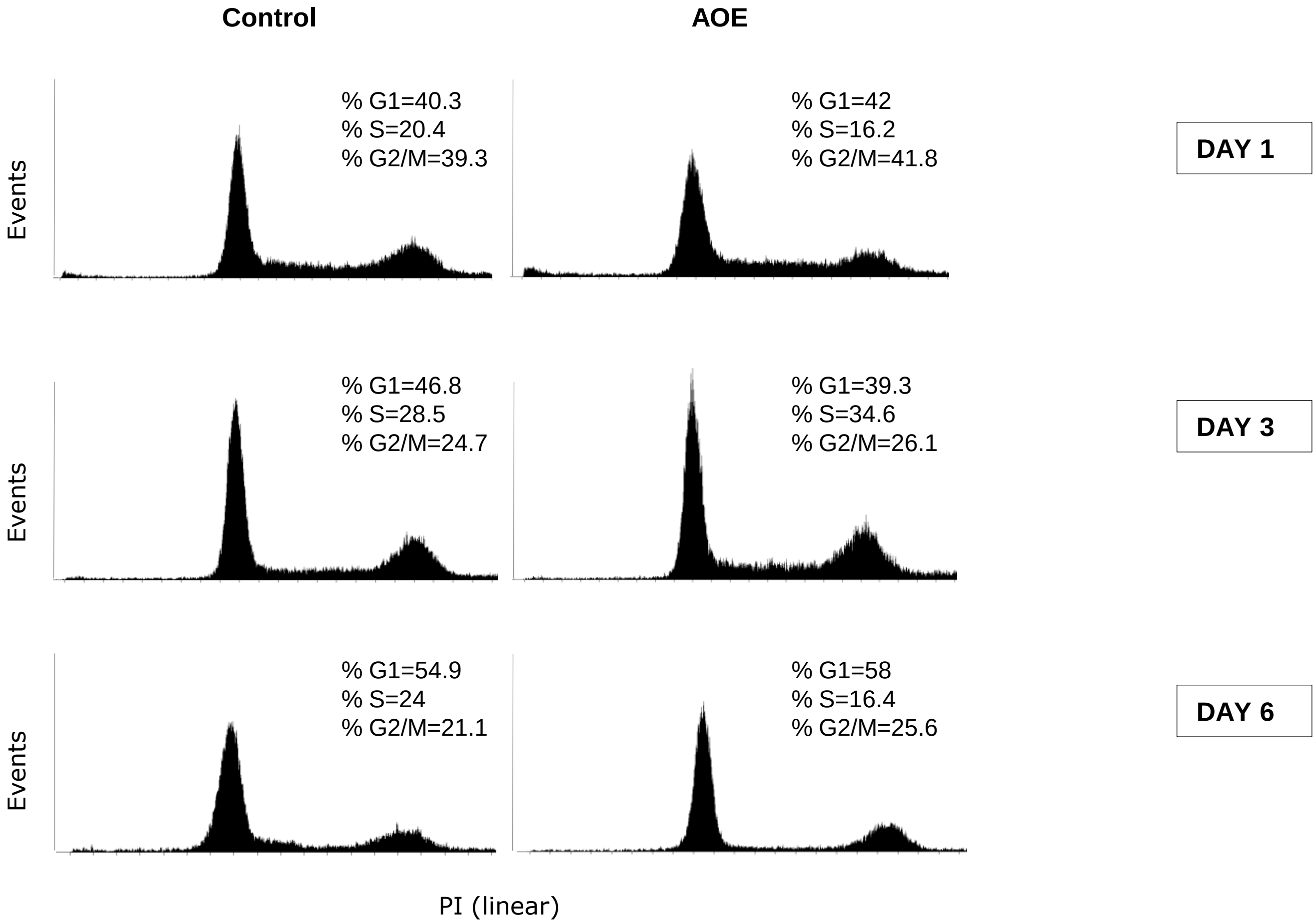
Control
AOE
% G1=40.3
% S=20.4
% G2/M=39.3
% G1=42
% S=16.2
% G2/M=41.8
DAY 1
% G1=46.8
% S=28.5
% G2/M=24.7
% G1=39.3
% S=34.6
% G2/M=26.1
DAY 3
% G1=54.9
% S=24
% G2/M=21.1
% G1=58
% S=16.4
% G2/M=25.6
DAY 6
Events
Events
Events
PI (linear)

Figure S5

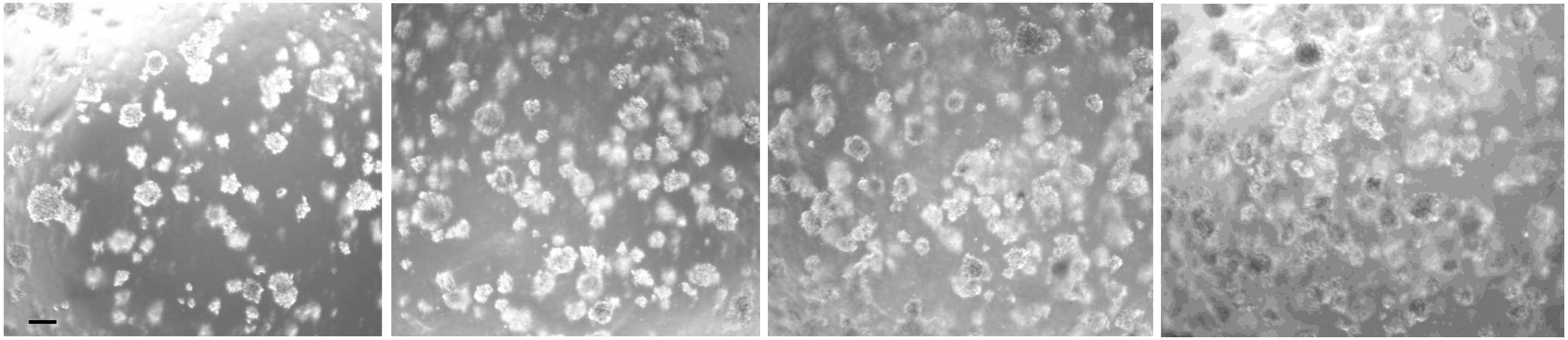

UN | 10 µl AOE | 50 µl AOE | 100 µl AOE
