# Supplementary material for: Epigenetic reprogramming of breast cancer cells with oocyte extracts
Source: Mol Cancer. 2011 Jan 13;10:7. doi: 10.1186/1476-4598-10-7 (PMC3034708; doi:10.1186/1476-4598-10-7)
Supplement: Additional file 5 — Q-PCR assay ID, primers and probes. The Table S1 lists the TaqMan gene expression assays, primers and probes used in this study. [file 1476-4598-10-7-S5.PDF]

Table S1: Q-PCR assays, primers and probes

	Assay ID	Forward	Reverse	Probe
**Q-PCR assay**				
RARB	Hs00977140_m1			
CST6	Hs00154599_m1			
GAS2	Hs00169477_m1			
CCND2	Hs00153380_m1			
ST18	Hs00608494_m1			
SRBC	Hs00293638_m1			
SCGB3A1	Hs00369360_g1			
GSTP1	Hs00168310_m1			
RASSF1A	Hs00168310_m1			
CDKN2A	Hs00233365_m1			
ACTB	Hs99999903_m1			
**ChIP**				
RARB		GGTAGGGTTCACCGAAAGTTCAC	AGCTCACTTCCTACTACTTCTGTCA	TCGCATATATTAGGCAATTCA
GAS2		TGGACACTGATTAACTGCAGCT	CGCAGTTAGAGGAAAGCATTCC	CCCAGCCTGTCTTTG
CDKN2A		CCAGCACCGGAGGAAGAAA	CGCCCCACCCTCTGG	CAGCCAGCCCCTCCTC
**Bisulfite sequencing**				
RARB		AGAGGTAGGAGGGTTTATTTTTTGT	TTTCAATTACATTTTCCAAACTTACTC	
CST6		GGTTTTTTGGGTTTTTTGAATTT	CTACCCATATTATAACTAACC	
CDKN2A		GAGGGGTTGGTTGGTTATTAGAG	TACAAACCCTCTACCCACCT	
CCND2		TTTGGAGTGAAATATATTAAAGGG	ATACAACTTTCTAAAAAATAACCC	
RASSF1A		GGGGGAGTTTGAGTTTATTGAGT	AACTCAATAAACTCAAACTCCCC	
ST18		AGAGGGTTGTTTTTTGATAGATATT	CAAAACTCTTTTCACTAAAAATAAAC	
OCT-4		ATTTGTTTTTTGGGTAGTTAAAGGT	CCAACTATCTTCATCTTAATAACATCC	
NANOG		TGGTTAGGTTGGTTTTAAATTTTTG	AACCCACCCTTATAAATTCTCAATTA	
